# Cocaine self-administration in adult female and male rhesus monkeys: longitudinal comparison with adolescent behavior and role of early life stress

**DOI:** 10.1038/s41386-025-02161-9

**Published:** 2025-07-05

**Authors:** Mia I. Allen, Erin R. Siebert, Alison G. P. Wakeford, Kendra Jenkins, Jessica Khan, Leonard L. Howell, Mar M. Sanchez, Michael A. Nader

**Affiliations:** 1 Department of Translational Neuroscience and the Center for Addiction Research, Wake Forest University School of Medicine, Winston-Salem, NC USA; 2 Emory National Primate Research Center, Emory University, Atlanta, GA USA; 3 Department of Psychiatry, School of Medicine, Emory University, Atlanta, GA USA

**Keywords:** Reward, Experimental organisms

## Abstract

A phenomenon involving cocaine use disorders is the “incubation of drug craving” - the drive for the drug increases the longer the abstinence period. The present longitudinal study provided a unique opportunity to test whether an increase in the reinforcing effects of cocaine developed after prolonged abstinence and if early life stress was a risk factor. Fourteen (*N* = 6 female, 8 male) adult rhesus monkeys, some (*N* = 7) that were maltreated as infants by their mothers (MALT), had previously self-administered cocaine under a fixed-ratio (FR) schedule of reinforcement as adolescents, but had not been studied for >3 years. In Experiment 1, cocaine self-administration dose-response curves were redetermined in adulthood when responding was maintained under the identical FR 20 schedule used during adolescence. In Experiment 2, the reinforcing strength of cocaine was evaluated (*n* = 12) under a progressive-ratio (PR) schedule of reinforcement. While there were no statistical differences between male and female monkeys on FR responding in adolescents, when redetermined as adults, MALT monkeys showed higher peak response rates relative to adolescence. No such differences were noted in Control monkeys. Under the PR schedule, peak reinforcing strength was not different between groups or sexes. However, higher total adolescent cocaine intake was significantly associated with higher cocaine breakpoints in adulthood. These findings show that after adolescent cocaine self-administration and a long abstinence period, sensitivity to cocaine reinforcement increased, particularly in monkeys who experienced early life stress. Although early life stress (MALT) did not significantly impact measures of cocaine’s reinforcing strength, higher adolescent cocaine intake did.

## Introduction

The prevalence of substance use disorders (SUDs), including cocaine use disorders (CUDs), continues to grow annually in the United States [1, 2]. In fact, the increase in cocaine use and related harms (i.e., overdose) has been recently termed the ‘silent’ epidemic since its impact has been largely muted by the unprecedented opioid crisis [3]. While cocaine misuse is a major public health problem, there are currently no FDA-approved pharmacotherapies for CUDs [4]. To aid in the development of novel treatments for CUDs, studies need to be conducted to identify factors that increase the probability of relapse to cocaine self-administration after abstinence or impact the risk of continued cocaine use after initiation of use [5]. Clinical studies have suggested that over the first few weeks of withdrawal from cocaine, human cocaine users become sensitized to cocaine-associated environmental cues that act as external stimuli for ‘craving’ [6–8]. This sensitization ultimately increases the risk of relapse [9]. Moreover, since adolescence is thought to be a period of greater vulnerability to cocaine use, heavy use during adolescence may predict poorer outcomes in terms of cocaine use in adulthood [10].

One phenomenon involving CUDs is the “incubation of craving” - the drive for drug seeking increases the longer the abstinence period [11]. Preclinically, evidence of this behavioral phenomenon has been well-documented in rodents, but not fully characterized in monkeys, particularly from a developmental perspective where cocaine initiation and chronic use is tested during adolescence, followed by prolonged abstinence and re-exposure to cocaine in adulthood. Moreover, although most preclinical studies have examined increases in the drive for drug seeking after an abstinence period using cue-induced reinstatement and extinction responding paradigms, few studies have examined this phenomenon in the context of changes in the reinforcing effects of drugs using self-administration procedures following prolonged abstinence [12, 13]. The female and male rhesus monkeys used in this study were unique in that they self-administered cocaine as adolescents [14, 15] and were not re-exposed to cocaine until adulthood (the present study). As a result, the present study provided an excellent opportunity to investigate whether the reinforcing effects of cocaine change after prolonged time off from cocaine in a within-subjects design.

Adolescence is a critical developmental phase for drug initiation since there is strong neurobiological remodeling during this time [16]. During this sensitive window, drugs can lead to perturbations to normative development and alterations in brain circuits critical for reward processing and stress/emotional regulation. This can potentially result in an increased susceptibility to SUDs and CUDs [16, 17]. Drug initiation during adolescence is a known risk factor for the development of drug dependence later in life [10, 18]. However, the neurobiological mechanisms underlying the increased risk during this developmental period are poorly understood, and studies have mainly depended on preclinical models using drug self-administration paradigms in rodents [19–23]. To date, the vast majority of adolescent rodent self-administration work has suggested that measures of adolescent drug self-administration typically exceed adult drug self-administration and that females show a unique susceptibility to psychostimulants as well as other drugs of misuse [19–23]. Together, these data demonstrate the importance of examining factors such as age and sex in terms of cocaine reinforcement. There are many fewer adolescent stimulant self-administration studies in nonhuman primates [14, 15, 24]. In fact, no studies to our knowledge, have directly compared adolescent versus adult self-administration in nonhuman primates across sexes.

Another factor that has not been thoroughly researched in nonhuman primates is the role of early life stress [25]. Among social, environmental, and genetic factors that increase an individual’s risk of SUDs, early life stress experiences significantly increase vulnerability for substance use [26–29]. Early life stress includes adverse experiences such as childhood maltreatment, and data support the premise that these experiences can have robust and long-lasting effects on numerous developmental outcomes, including risk of substance use (e.g., [26]). Specifically, there have been reports in the literature showing that early life stress experiences can lead to an increased risk for SUDs and worse outcomes, including severity of drug use as well as propensity to relapse following abstinence. Sex differences have also been noted [27, 28]. In animal models, our group has studied the role of early life stress on cocaine reinforcement using a translational rhesus monkey model of infant maltreatment by the mother (MALT). Using this model, we previously reported long-term alterations in neurobehavioral development, specifically, increased anxiety and emotional reactivity, impaired impulse control, hyperactivity of stress neuroendocrine systems [30–32], and alterations in prefrontal, amygdala, and reward circuits, including dopamine and serotonin function [33–35]. The female and male rhesus monkeys used in this study were part of those longitudinal examinations.

Previous work in the cohort of male and female rhesus monkeys used in the current study has also demonstrated that when cocaine was available under a fixed-ratio (FR) 20 schedule of reinforcement during adolescence, MALT males showed faster acquisition (required less sessions) to acquire cocaine self-administration when compared to control males and all female groups (Control, MALT). However, in general, no significant differences in rates of responding as a function of cocaine dose were observed between MALT male and female monkeys and age-matched control monkeys. When the cocaine dose that resulted in peak response rates was studied for 25 consecutive sessions, males (both MALT and Controls) had significantly higher rates of responding compared with females [36]. A follow-up study in these monkeys compared cocaine self-administration during 4-hr extended access to 1-hr limited access, with no evidence of escalation of cocaine intake and no evidence of differences between MALT and Controls [15]. Overall, these findings suggest that early life stress may confer enhanced sensitivity to the reinforcing effects of cocaine, especially in males, but only under certain conditions [14].

Following these initial assessments during post-pubertal adolescence, the monkeys underwent a prolonged abstinence period (>3 years) prior to re-assessment of cocaine self-administration in adulthood in this study. As a result, the present study was well positioned to address questions related to the incubation of cocaine ‘craving’ following extended time-off from cocaine self-administration. As noted above, incubation of cocaine ‘craving’ has been proposed as a time-dependent increase in cue-induced cocaine ‘craving’ during prolonged periods of cocaine deprivation [36, 37]. Although ‘craving’ can be assessed in many ways in clinical research, it is impossible to directly measure ‘craving’ in animal models [38]. The present study provided a novel opportunity to test the phenomenon that the reinforcing effects of cocaine can change following protracted abstinence from a developmental longitudinal perspective and in the context of early life stress and sex as a risk factor. Finally, we extended these FR self-administration studies to include progressive-ratio (PR) responding to examine whether higher levels of cocaine intake during adolescence would increase the reinforcing strength of cocaine in adulthood, particularly in individuals with early life stress.

## Methods

### Subjects

A total of 14 (*n* = 7 Controls: 4 males, 3 females; *n* = 7 MALT: 4 males, 3 females) adult rhesus macaques (*Macaca mulatta*) were used in these studies. All monkeys had a previous history of i.v. cocaine self-administration during adolescence under an FR 20 schedule of reinforcement [14, 15] but had not self-administered cocaine for a prolonged period of time ranging from 3.1-4.8 years (Supplementary Table 1). A two way-ANOVA evaluating significant differences in the length of abstinence as a function of sex or rearing condition (Controls vs MALT) found no significant differences (*p* > 0.05). Catheters were occasionally flushed to maintain patency, but no operant self-administration studies were conducted. While some of the monkeys (Ctrl-F-3, Ctrl-M-1, MALT-M-6, Ctrl-M-3, MALT-M-2) were able to retain the same catheters during the adolescent and adult cocaine dose-response curve determinations, others (MALT-F-2, MALT-F-3, MALT-F-4, Ctrl-F-1, Ctrl-F-2, MALT-M-1, MALT-M-3, Ctrl-M-5, Ctrl-M-2) had their catheters removed and re-implanted due to loss of patency, between cocaine dose-response curve determinations. These monkeys were born and raised with their mothers and families in large social groups at the Emory National Primate Research Center (ENPRC) and were ~4.5–6.7 years old when first exposed to cocaine in adolescence and ~11–13 years old at the start of this study in adulthood (Supplementary Table 1). Seven monkeys (*n* = 3 females and *n* = 4 males) were maltreated by their mothers as infants (MALT; see [14, 39]) and seven (*n* = 3 females and *n* = 4 males) received competent maternal care (Controls); all have been studied longitudinally as part of a larger project examining the developmental consequences of infant MALT from birth into adulthood [14, 15, 25, 30–32, 34, 35]. In this early life stress model, infant MALT is defined as comorbid maternal physical abuse and rejection of the infant during the first 3-6 months of life (equivalent to 12-24 months in humans), which causes distress and elevations in stress hormones [30, 31].

At approximately 4-5 years of age, animals were transferred from the ENPRC Field Station to the ENPRC Main Station. With the exception of Malt-F-2 and Ctrl-F-1, who were pair-housed, all monkeys in this study were individually housed in temperature and humidity-controlled, same-sex rooms, maintained on a 12-h light/dark cycle (lights on between 7:00 AM and 7:00 PM), in stainless steel cages with *ad libitum* access to water. The facilities were licensed by the United States Department of Agriculture and accredited by AAALAC International. At the start of the present study, monkeys were weighed weekly and fed standard chow daily (Purina Mills Int., Lab Diets, St. Louis, MO), which was also supplemented with fresh fruits and vegetables. Monkeys were fed chow after self-administration sessions. Monkeys were not food restricted; chow amounts were determined by veterinary staff to keep monkeys at a healthy weight, which was monitored monthly throughout the study. Body weights did not vary significantly between the adolescent cocaine dose-response curve determination (M = 10.37, SD = 2.12) and the adult cocaine dose-response curve determination (M = 9.45, SD = 1.98) (*p* > 0.05). Correlations showed that body weights did not predict any of the main outcomes in this study (*p* > 0.05). Environmental enrichment was provided in the home cage on a regular basis. Research and husbandry were conducted in accordance with the Animal Welfare Act and the U.S. Department of Health and Human Services 2011 Guide for the Care and Use of Laboratory Animals. The Emory University Institutional Animal Care and Use Committee approved the research protocol.

### Catheters

Under sterile conditions, monkeys were surgically implanted with a chronic indwelling intravenous catheter and a subcutaneous vascular access port (VAP; Access Technologies, Skokie, IL, USA), as previously described [40]. Each animal received a pre-operative antibiotic (30 mg/kg Kefzol, i.m.; cefazolin sodium, Marsam Pharmaceuticals Inc., Cherry Hill, NJ, USA). Ketamine (10–15 mg/kg, i.m.) and Telazol (2 mg/kg i.m.) were used to sedate animals, and anesthesia was maintained during the procedure with 1-1.5% isoflurane gas. Surgical details can be found in previously published work [41]. During post-operative recovery, monkeys received either ketoprofen (5 mg/kg, i.m.) or Metacam (meloxicam; 1.5 mg/kg, p.o.), and starting the following day, Naxcel (ceftiofur sodium; 2.2 mg/kg, i.m., SID) for 7–14 days. Monkeys were not studied for at least 7 days post-surgery. Each port and catheter was flushed with a heparinized saline solution (100 U/mL) between sessions to prolong patency and minimize clotting. When catheter patency issues were suspected, catheters were determined to be patent with the occurrence of visible ataxia following intravenous administration of methohexital sodium (3.0 mg/kg i.v.).

### Apparatus

All monkeys were fitted with plastic or aluminum collars. Following this, they were trained to sit in a primate restraint chair (Primate Products, Miami, FL, USA) and habituated to sound-attenuating, ventilated, operant chambers that were fitted with a panel that consisted of one response lever and stimulus lights. The needle of a right-angle Huber infusion set (Access Technologies, Skokie, IL) was inserted into the indwelling VAP. The infusion set Tygon tubing was connected to the drug syringe in a motor-driven syringe pump (Harvard Apparatus PhD 2000); cocaine solution was located outside of the chamber (Med Associates, St. Albans, VT). The syringe pump delivered a volume of 0.5 ml/infusion over 3 s.

Prior to each session, the area on the monkey’s back surrounding and including the VAP was prepared alternating two rounds of isopropyl alcohol pads (Fisher Scientific, Fair Lawn, NJ, USA) and Chloraprep Applicators (VWR) before a 22-gauge Huber needle (Access Technologies) was inserted into the monkey’s VAP site, connecting the catheter to the syringe infusion pump on the side of the chamber. Before each session, the pump was operated for 3 seconds, dispensing 0.5 ml, to fill each monkey’s port with either saline or the concentration of cocaine available.

### Procedure

#### Experiment 1. Examining the effects of time-off from cocaine on the rates of self-administration under a fixed-ratio (FR) schedule of reinforcement between adolescence and adulthood

**Fig. 1:**
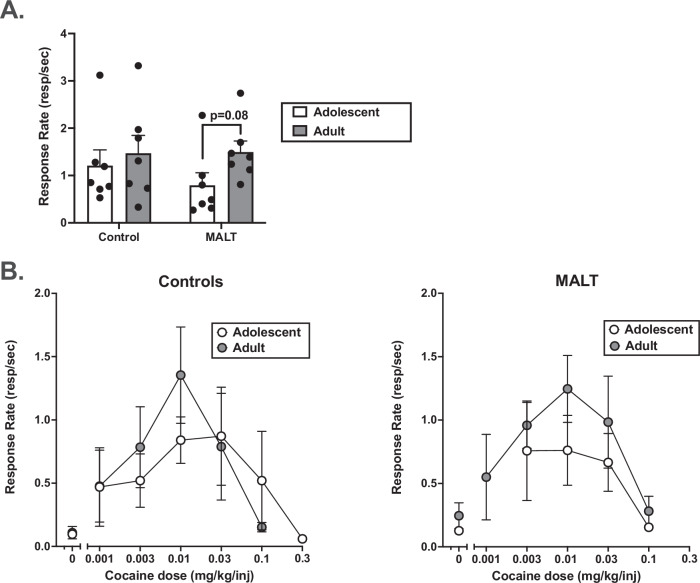
Average cocaine response rates at peak dose and dose-response curves in Control and MALT monkeys across adolescence and adulthood. **A** Average cocaine response rates (resp/sec), irrespective of sex, at the peak of the cocaine dose-response curve in Control and MALT monkeys during the initial determination in adolescence (white bars) and during the second determination in adulthood (black bars). **B** Averaged dose-response curves, irrespective of sex, as a function of group (Control on the left, MALT on the right). Data represent the mean ± the standard error of the mean. *N* = 14 (*n* = 7 Controls, *n* = 7 MALT).

**Fig. 2:**
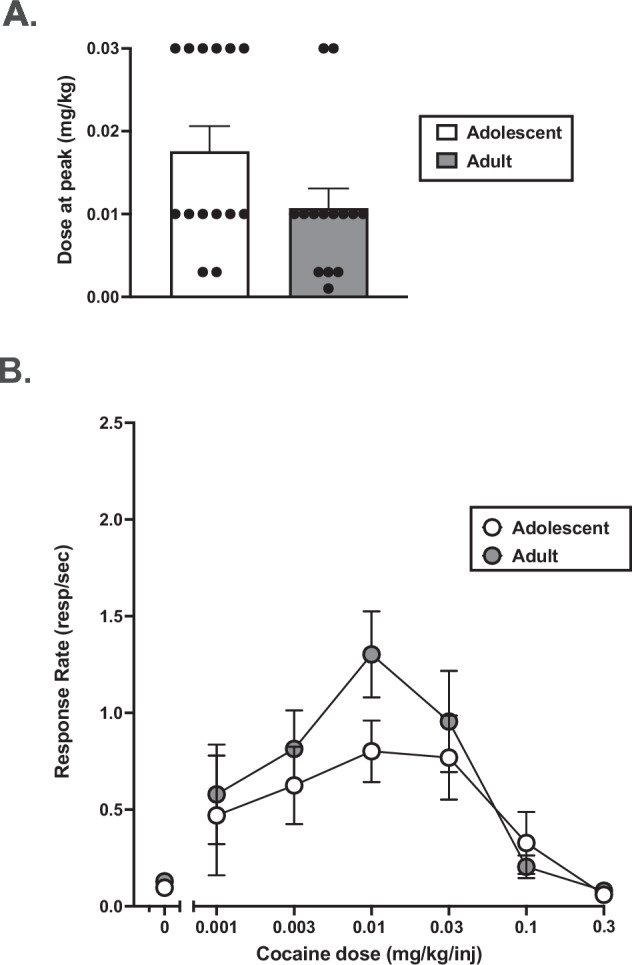
Average cocaine dose producing peak response rates and dose-response curves irrespective of group and sex in adolescence and adulthood. **A** Irrespective of group and sex, average cocaine dose (mg/kg) at the peak of the self-administration dose-response curve during the initial determination in adolescence and during the second determination in adulthood. **B** Averaged dose-response curves across sex and group. Data represent the mean ± the standard error of the mean. *N* = 14 (*n* = 7 Controls, *n* = 7 MALT).

At the start of this experiment, monkeys had not been studied for ~3 years; the initial dose of cocaine available was the lowest dose that resulted in response rates higher than saline-contingent rates when the cocaine dose-response curve was first determined (see Figs. 1 and 2, open symbols) Responding was re-established by cocaine under low FR values (1–10) and gradually increased over 1-3 sessions until the final FR 20 was established. After stable responding under this saline and each cocaine dose (0.001–0.3 mg/kg/injection) was made available for at least 5 sessions and until responding was stable, defined as response rates not varying by more than 20% of the 3-day mean at each dose, with no overall upward or downward trend over the 3 days. Cocaine doses were tested in pseudo-random order in each monkey (the highest cocaine dose was never tested first).

#### Experiment 2. Assessment of the reinforcing strength of cocaine under a progressive-ratio (PR) schedule of reinforcement in adulthood

After Experiment 1, the conditions were changed to a progressive-ratio (PR) schedule of reinforcement, as previously described [41–44]. The first response requirement under the PR schedule was 20, and each subsequent cocaine injection was determined by the formula established by Richardson and Roberts [45]; there was a 10-second timeout after each drug injection. Sessions ended after a maximum of 20 injections, the 60-minute limited hold expired, or 4 hours had elapsed. The limited hold corresponded to the maximum time allotted for monkeys to receive an injection of cocaine. The maximum number of injections earned at each dose was termed the breakpoint (BP). For all monkeys, the initial cocaine dose was 0.03 mg/kg/injection since this dose was generally found at the peak of cocaine dose-response curves in monkeys. Full cocaine dose-response curves (0.001–0.3 mg/kg/injection) were determined in each monkey, and doses were tested in pseudo-random order. Saline was also made available. Between dose changes, each subject ran for two days on a 0.03 mg/kg/injection session. Each drug dose was kept constant for at least 3 sessions and until stable, defined as the number of injections not varying by more than 20% of the 3-day mean at each dose, with no overall upward or downward trend over the 3 days.

### Drugs

The National Institute on Drug Abuse (Bethesda, MD) supplied cocaine, which was dissolved in 0.9% sterile saline. All drug doses are expressed as the salt form.

### Data analyses

For both experiments, individual-subject data are shown as means ± SD of the last three sessions, and group data are shown as mean ± SEM. For Experiment 1 (FR), the primary dependent variable was response rates (responses per second). Paired t-tests were used to determine whether a cocaine dose was reinforcing by comparing cocaine-maintained responding to response-dependent saline injections. Response rates at the peak of the dose-response curve for each individual monkey were compared using a mixed-effects ANOVA with sex and group (Control vs MALT) as between-subject factors and determination time (adolescence vs adulthood) as the within-subject factor. Moreover, a mixed-effects ANOVA using the same factors was conducted on the dose of cocaine that was at the peak of the dose-response curve for each individual monkey as a proxy of potency, since ascending dose ED_50_ values could not be calculated for all monkeys. If there were no statistically significant results (i.e., sex, group, determination time, or interaction effects were non-significant), repeated measures ANCOVAs were performed to investigate whether sex had a significant effect while controlling for group or if group had a significant effect while controlling for sex. An ANCOVA was used so that we could statistically control for the variance associated with either sex or group in the model. This increased statistical power and allowed us to investigate the role that sex or group had on the main dependent variables without added variance from other main factors. In addition, linear regressions were run to determine if adolescent cumulative cocaine intakes (mg/kg) (Supplementary Table 2) predicted cocaine peak response rates and the peak dose of cocaine from the adult-determined dose-response curve. Sex and group were initially included in all these models, but were removed if they were not significant predictors.

For Experiment 2 (PR), the primary dependent variable was break point (BP) or the maximum number of injections earned at each dose. Paired t-tests were used to determine whether a cocaine dose was reinforcing by comparing cocaine BPs to saline BPs. The mean number of infusions (i.e., BP) was plotted as a function of dose, and peak BPs were compared using a two-way ANOVA as a function of group (Control vs MALT) and sex. In the absence of statistically significant results, one-way ANCOVAs were run while controlling for either group or sex. An ANCOVA was selected for the same reasons noted above. Moreover, simultaneous linear regressions were run to determine if adolescent cumulative cocaine intakes (mg/kg) predicted cocaine BPs during adulthood (Supplementary Table 2 and 3). These adolescent cumulative cocaine intakes (mg/kg) were taken following the adolescent cocaine dose-response curve determination and ranged from 40–415 mg/kg. There was a range based on several factors including how many days it took the monkey to reach the stability criterion at each dose, which doses needed to be tested based on the shape of the dose-response curve, variability in the number of injections each monkey received at various doses, and whether they were tested in cocaine extended access conditions. This analysis was conducted to determine whether higher adolescent exposure to cocaine was related to the reinforcing strength of cocaine in adulthood. Monkeys Ctrl-F-1 and MALT-M-3 were not included in these analyses since these monkeys did not complete a PR cocaine dose-response curve in adulthood. Sex and group were initially included in these models but were removed if they were not significant predictors. For both experiments, the Sidak correction was used to correct for multiple comparisons. Significance was set at an alpha less than 0.05, and all statistical tests were analyzed with SPSS.

## Results

### Experiment 1. Examining the effects of time-off from cocaine on the rates of self-administration under a fixed-ratio (FR) schedule of reinforcement between adolescence and adulthood

In all monkeys during both adolescent and adulthood determinations (Fig. 1), cocaine functioned as a reinforcer in that response rates were significantly higher for at least one dose of cocaine when compared to response rates when saline was available (*p* < 0.05) (Supplementary Fig. S1A and S1B). A mixed-effects ANOVA using individual dose-response curves (Supplementary Table 2 and Supplementary Fig. S1A and S1B) found no significant effect of determination time (adolescent vs adulthood) (F(1,10) = 2.42, *p* = 0.151), or interaction between sex and determination time (F(1,10) = 0.711, *p* = 0.419), group and determination time (F(1,10) = 0.761, *p* = 0.555), or three way interaction (F(1,10) = 0.045, *p* = 0.836) on cocaine response rates at the peak of the dose-effect curves. When group was used as a covariate, there was no significant effect of determination time (F(1,11) = 0.236, *p* = 0.637), or interaction between sex and determination time (F(1,11) = 0.779, *p* = 0.396) on cocaine response rates at the peak of the dose-response curves (Supplementary Fig. S2A and S2B). When sex was added as a co-variate, there was a trending interaction between determination time and group (F(1,11) = 3.67, *p* = 0.08) such that in the MALT monkeys, peak cocaine response rates were higher during adulthood (M = 1.60, SEM = 0.305) when compared to adolescence (M = 0.787, SEM = 0.293) (Fig. 1A and B). No such difference was found in the Control monkeys (F(1,11) = 0.335, *p* = 0.575). Visual inspection of individual dose-response curves (Supplementary Fig. S1) showed that while only 3/7 Control monkeys had higher response rates during adulthood when compared to adolescence (panels A and B), in MALT monkeys all but one monkey had higher response rates during adulthood when compared to adolescence (panels C and D). Linear regressions demonstrated that cumulative cocaine intakes during adolescence (Supplementary Table 2**)** did not predict peak cocaine response rates during adulthood (t(12) = −1.59, *p* = 0.140).

A mixed-effects ANOVA also found no effect of determination time (F(1,10) = 2.87, *p* = 0.121), interaction between sex and determination time (F(1,10) = 0.284, *p* = 0.606), or interaction between group and determination time (F(1,10) = 0.467, *p* = 0.510) on the dose of cocaine that was at the peak of the dose-response curve (Fig. 2A and B). When group was held constant, there was no effect of determination time (F(1,11) = 3.18, *p* = 0.102), or interaction between sex and determination time (F(1,11) = 0.311, *p* = 0.588), on the dose of cocaine that was at the peak of the dose-response curve. When sex was used as a co-variate, there was also no effect of determination time (F(1,11) = 3.17, *p* = 0.102), or interaction between group and determination time (F(1,11) = 0.551, *p* = 0.474), on the dose of cocaine that was at the peak of the dose-response curve. Linear regressions demonstrated that cumulative cocaine intakes during adolescence (Supplementary Table 2**)** did not predict the dose at the peak of the cocaine dose-response curves during adulthood (t(12) = 1.21, *p* = 0.252).

#### Experiment 2. Assessment of the reinforcing strength of cocaine under a progressive-ratio (PR) schedule of reinforcement in adulthood

**Fig. 3:**
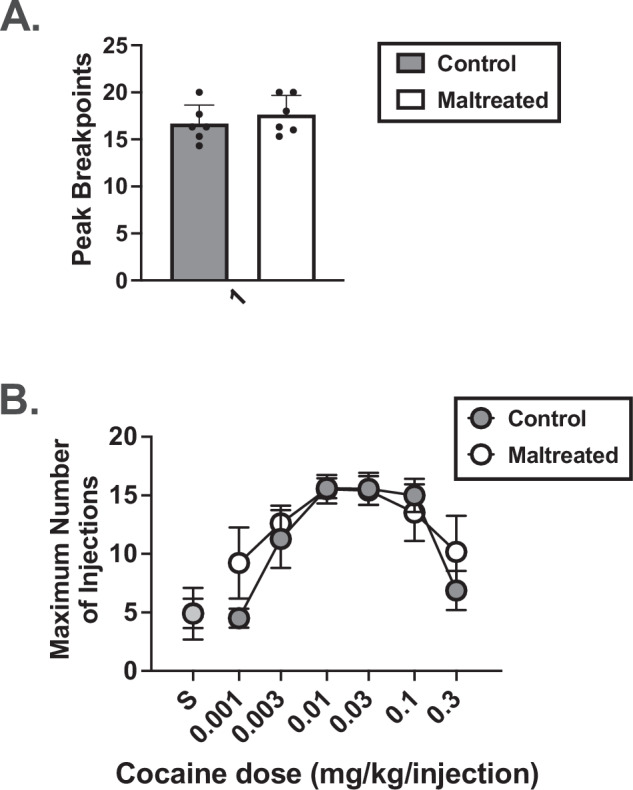
Average cocaine BPs and dose-response curves in Control and MALT monkeys during adulthood. **A** Average peak cocaine BPs (maximum number of injections) during adulthood as a function of group. **B** Averaged cocaine dose-response curves as a function of group. Data represent the mean ± the standard error of the mean. *N* = 12 (*n* = 6 Controls, *n* = 6 MALT).

**Fig. 4:**
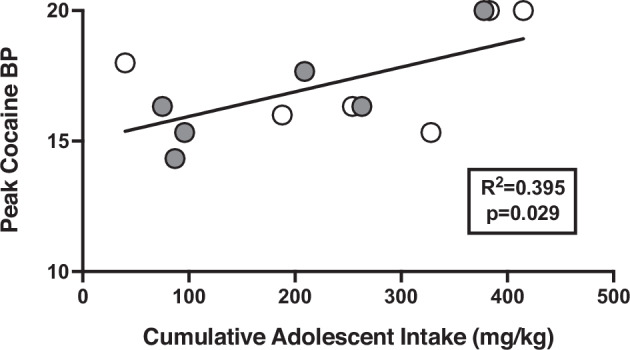
Relationship between adolescent cocaine intakes and peak cocaine breakpoints in adulthood. Regression plot depicting a regression line between peak cocaine BPs (maximum number of injections) during adulthood and cumulative cocaine intakes (mg/kg) during adolescence. The doses used on the abscissa were individually determined. Adolescence cocaine intake was a significant predictor of peak cocaine BP during adulthood. *N* = 12 (*n* = 6 Controls, *n* = 6 MALT).

In all monkeys, cocaine BPs were significantly higher than saline BPs demonstrating that cocaine functioned as a reinforcer (Supplementary Fig. S3A and S3B). Cocaine BPs and dose at which the peak BP occurred in individual subjects can be seen in Supplementary Table 3. When both sex (F(1,12) = 0.004, *p* = 0.951) and group (F(1,12) = 0.418, *p* = 0.536) were included in the model there were no significant differences in the cocaine dose associated with peak BPs (Fig. 3A). When group or sex were held constant, neither variable had a significant effect on cocaine BPs (*p* > 0.05). As a result, averaged dose-response curves in all animals regardless of sex or condition are shown in Fig. 3B. To assess whether higher adolescent cocaine intakes would relate to cocaine having a higher reinforcing strength in adulthood (i.e., higher BPs), linear regressions were run. These demonstrated that adolescent cocaine intake was a significant positive predictor of cocaine BPs in adulthood (t(11) = 2.56, *p* = 0.029, r^2^ = 0.395) such that higher adolescent cocaine intakes were associated with higher cocaine BPs during adulthood (Fig. 4**)**. Neither sex nor group were significant predictors in this model (*p* > 0.05).

## Discussion

Previous clinical research has shown that over the first few weeks of cocaine abstinence, cocaine users become sensitized to cocaine-associated environmental cues that increase ‘craving’, preclinically described as “incubation of cocaine craving”. While there is evidence of “incubation” in rodents [36], there is not in nonhuman primate models. However, incubation studies typically involve responding leading to a stimulus associated with cocaine, rather than self-administering cocaine. In studies testing the premise that abstinence-induced craving leads to increases in drug use, there is some evidence of increases in the reinforcing effects of cocaine in humans, but short-term time off from cocaine in monkeys did not result in higher rates of cocaine self-administration [6, 46, 47]. Since cocaine abstinence may play an important role in the risk of relapse, characterizing how extended time-off from cocaine may modulate later reinforcing effects of cocaine is crucial for the evaluation of behavioral and pharmacological interventions, particularly if we want to understand how cocaine initiation during adolescence affects risk for cocaine use during adulthood and following prolonged abstinence periods.

The present study provided a unique opportunity to address these questions from a developmental longitudinal perspective and in the context of early life stress as a risk factor. In the present study, the reinforcing effects and the reinforcing strength of cocaine were studied in adult female and male rhesus monkeys with and without early life stress (MALT by their mothers as infants) and compared with cocaine self-administration data collected during adolescence. Redetermining the cocaine dose-response curves under the same fixed-ratio schedule, but after a prolonged (>3 years) abstinence period, found greater increases in cocaine sensitivity in MALT monkeys from both sexes when compared with controls. When the reinforcing strength of cocaine was studied under a progressive-ratio schedule, no group or sex differences were observed. However, the amount of cocaine used during adolescence was significantly associated with peak breakpoints, suggesting that an adolescent history of cocaine use can influence later adult vulnerability, especially after long periods of abstinence.

### Changes in cocaine self-administration vary based on early life stress

The primary goal of the present study was to extend earlier findings on the long-term impact of early life stress on cocaine self-administration. Initial studies from our group noted only modest effects of infant maltreatment on rates of cocaine self-administration under a fixed-ratio schedule of reinforcement during adolescence [13, 36]. The first question addressed in the present study was whether extensive time-off from cocaine would enhance the reinforcing effects of cocaine in adulthood and if there were group and sex differences. While several studies using rodents have shown that time off from cocaine self-administration increased cue-induced responding [37, 48–51], only one previous study examined this phenomenon in rhesus monkeys, and that was up to 14 days off, with no effects noted [47]. The present study not only studied cocaine self-administration after a longer period of time off from cocaine (~3 years), but also examined how adolescent maltreatment and cocaine use would impact the reinforcing effects of cocaine in adulthood.

Few preclinical studies have examined the effects of stress on the incubation of cocaine ‘craving’, and those that have primarily focused on whether stress during time-off from cocaine intensifies the incubation of cocaine craving [52]. For instance, one study found that rats exposed to repeated restraint stress during early times off from cocaine had an enhanced rate of incubation of cocaine craving when compared to controls [53]. To our knowledge, though, no preclinical studies have directly examined how early life stress influences the trajectory of changes in the reinforcing effect of cocaine after protracted abstinence. However, clinical studies have demonstrated that patients who experienced early life stress relapse more often, remain abstinent for shorter periods of time, and are less responsive to treatments [54]. Moreover, one study found that women who were in early abstinence from crack cocaine had higher craving levels that lasted longer if they had a history of childhood physical neglect [55]. The results of this study provide further support for this relationship and suggest that early life stress can potentiate changes in cocaine reinforcement after abstinence.

Contrary to the premise associated with incubation of craving, it seems intuitive that the longer an individual is abstinent from cocaine, the more likely they will remain cocaine free. One way to evaluate changes in sensitivity to cocaine reinforcement is to examine the potency of cocaine at the peak of the self-administration dose-response curve. Irrespective of whether exposed to early life stress and the sex of the monkey, there were no apparent differences in sensitivity between adolescent and adult cocaine self-administration. That is, the dose at the peak of the dose-response curve was not different in adolescents and adults, using a within-subjects design. However, although not statistically significant, MALT monkeys showed a trend towards higher peak response rates during adulthood relative to data obtained as adolescents. This effect was not seen in the Control monkeys and did not vary based on sex.

### Changes in cocaine self-administration do not vary based on sex

Preclinical studies examining sex differences in the incubation of cocaine ‘craving’ have yielded mixed results but, unlike the current study, most recent findings have shown the female rats, relative to males, have greater increases in the number of active lever responses on a fixed-ratio schedule of reinforcement following time-off from cocaine [56, 57]. No studies in monkeys have examined sex differences in relation to the effect of time-off on cocaine reinforcement [47]. Clinically, reports have been mixed, and there is little available evidence on the incubation of drug craving differing as a function of sex due to limited sample sizes in each sex [50]. As noted above, preclinical studies examining incubation of cocaine craving have examined time-dependent changes in cocaine-cue responding rather than changes in cocaine self-administration following prolonged time off from cocaine [12]. These different experimental approaches could, in part, explain why this study did not find sex differences while other studies have. In fact, it is possible that the behavioral and neurobiological mechanisms underlying cocaine seeking, typically assessed via extinction-reinstatement paradigms, and cocaine self-administration may not align. As a result, the findings of this study may not be generalizable to studies examining cue-induced reinstatement after protracted time off from cocaine.

### Changes in cocaine self-administration under PR schedule relate to adolescent cocaine intakes

The present study also extended the evaluation of infant maltreatment on later measures of cocaine self-administration to include an assessment of reinforcing strength using a progressive-ratio schedule of reinforcement. Similar to fixed-ratio responding in Experiment 1, peak breakpoints for cocaine did not differ between adult males and females of either group. This finding is inconsistent with a study in rodents showing that following at least one week of deprivation from cocaine and *d*-amphetamine there were significant increases in reinforcing strength under a progressive-ratio schedule of reinforcement [58]. It is not clear if the difference is due to the substantially longer history of cocaine self-administration in monkeys from the present study, or to the fact that the rodent study involved a redetermined break point, while this was the monkeys first exposure to the progressive-ratio schedule.

While sex and MALT did not appear to influence breakpoints, cumulative adolescent cocaine intakes predicted higher reinforcing strength of cocaine during adulthood. This also occurred independent of sex or early life stress exposure. One possibility is that exposure to cocaine in adolescents affected brain regions that mediate the reinforcing strength of cocaine. PET imaging studies examining dopamine and serotonin receptor systems from these monkeys in adolescence (before cocaine self-administration) have been published [35] and we are currently comparing those data with adult PET imaging data. Several clinical studies have supported the notion that adolescence is a period of heightened vulnerability where adolescent cocaine use increases the likelihood of developing a CUD [10, 59]. Preclinically, these findings have been replicated, and adolescent cocaine exposure appears to modify the sensitivity of adults to the reinforcing effects of cocaine [60]. The imaging data, along with the present self-administration data, should provide valuable information to understand the long-term consequences of cocaine use, time off from cocaine, and re-exposure to cocaine.

An additional implication to the present findings involves the evaluation of behavioral and pharmacological interventions to decrease cocaine use. It is possible that, if another group of rhesus monkeys self-administered cocaine as adults, but did not have the adolescent cocaine exposure, their behavior would look like the monkeys in this study, but the efficacy of interventions would be different. Again, this likely outcome may be reflected in how the adult brain responds to cocaine and the impact of adolescent exposure. These are important empirical questions that may aid in the development of personalized medicine approaches to treating CUDs.

Finally, it is important to note that the effects of early life stress on measures of reinforcing effects and reinforcing strength of cocaine were negligible in the present study. As noted above, this should not be interpreted as early life stress not having effects on cocaine reinforcement, since we have not evaluated interventions or changes in the contingencies. For example, in socially housed male monkeys, subordinate animals self-administer cocaine at higher rates than dominant monkeys [61]. However, after extensive cocaine exposure, these differences are no longer apparent [62]. When the conditions were changed from an FR schedule to a concurrent cocaine vs. food choice contingency, subordinate male monkeys were again more sensitive to cocaine reinforcement [63]. Future research in these monkeys will be required to determine the long-term impact of adolescent cocaine exposure on current cocaine self-administration.

### Limitations

There are several limitations that should be noted. First, a limited number of monkeys were available for the current study, and thus, we were statistically underpowered for certain analyses. A second limitation was in Experiment 1, where food-maintained responding under a fixed-ratio schedule of reinforcement was not assessed during adolescence or during adulthood. Studies have shown that incubation of drug craving can also generalize to food-maintained responding, where both sucrose and saccharin self-administration were increased following a period of deprivation, even when rats had *ad libitum* access to food in the home cage [64, 65]. Future studies should investigate this possibility. Another limitation was that the cocaine dose-response curves were determined years apart, while most clinical studies examine the incubation of craving occur over a few weeks into cocaine abstinence [6, 46, 47]. Importantly, under the fixed-ratio schedule of reinforcement, changes in sensitivity to cocaine reinforcement from adolescence to adulthood in MALT could have occurred because of the long-term effects of adolescent cocaine exposure rather than the expression of incubation of cocaine seeking. Without a control group where cocaine dose-response curves were redetermined twice in adulthood, it is not possible to definitively conclude the cause of time-dependent changes in the potency of cocaine. Thus, the findings of this experiment should be interpreted in the context of this caveat.

Furthermore, because PR dose-response curves were not determined prior to time off from cocaine, no conclusions can be made about how extended time off may change the reinforcing strength of cocaine. However, since a previous study in monkeys found that there were no alterations in cocaine breakpoints after 3, 7, or 14 days off from cocaine under a progressive-ratio schedule of reinforcement, it is possible that deprivation from cocaine does not influence reinforcing strength [47], at least in adults and as assessed with progressive-ratio responding.

## Conclusions

This study demonstrated that following a long period of abstinence (>3 years) after an extensive history of cocaine self-administration in adolescence, sensitivity to cocaine reinforcement increased, particularly in MALT monkeys. Although sex did not significantly impact current measures of cocaine self-administration under FR or PR schedules of reinforcement, we found that higher adolescent cocaine intakes predicted higher reinforcing strength for cocaine in adulthood. Overall, these findings suggest that after an extensive history of cocaine use during adolescence and following a prolonged time off from cocaine, the reinforcing effects of cocaine may be enhanced, particularly in MALT monkeys. Ongoing research will aim to elucidate the neural underpinnings of these effects.

## Supplementary information

Supplementary Material

## Data Availability

Data will be available upon request.
