# Supplementary material for: Cocaine self-administration in adult female and male rhesus monkeys: longitudinal comparison with adolescent behavior and role of early life stress
Source: Neuropsychopharmacology. 2025 Jul 5;50(13):2006–14. doi: 10.1038/s41386-025-02161-9 (PMC12603046; doi:10.1038/s41386-025-02161-9)
Supplement: Supplementary file 1 — Supplementary Material [file 41386_2025_2161_MOESM1_ESM.pdf]

Supplemental File

Cocaine Self-Administration in Adult Female and Male Rhesus Monkeys: Longitudinal Comparison with Adolescent Behavior and Role of Early Life Stress

Mia I. Allen[1], Erin R. Siebert[2], Alison G.P. Wakeford[2], Kendra Jenkins[2], Jessica Khan[2], Leonard L. Howell[2,3], Mar M. Sanchez[2,3], Michael A. Nader[1]

[1]Department of Translational Neuroscience and the Center for Addiction Research, Wake Forest University School of Medicine, Winston-Salem, NC 27157

[2]Emory National Primate Research Center, Emory University, Atlanta, GA 30329

[3]Department of Psychiatry, School of Medicine, Emory University, Atlanta, GA 30322

Address Correspondence to:

Michael A. Nader, Ph.D.
Department of Translational Neuroscience
Wake Forest University School of Medicine
NRC 546, Medical Center Blvd.
Winston-Salem, NC 27157-1083
mnader@wakehealth.edu
336-713-7172

Supplemental Table 1. Monkey Groups and Histories

Monkey	1st DRC Age (yrs)	Time-Off (yrs)	2nd DRC Age (yrs)
**Control Females**			
**Ctrl-F-1**	6.71	4.58	13.16
**Ctrl-F-2**	4.55	3.19	11.00
**Ctrl-F-3**	4.53	3.11	10.98
**Mean**	5.26	3.63	11.71
**SEM**	0.88	0.58	0.88
**MALT Females**			
**Malt-F-2**	6.61	4.70	13.06
**Malt-F3**	5.70	3.56	12.15
**Malt-F4**	6.62	4.39	13.07
**Mean**	6.31	4.22	12.76
**SEM**	0.37	0.37	0.37
**Control Males**			
**Ctrl-M-1**	4.66	3.67	11.11
**Ctrl-M-2**	4.66	3.11	11.11
**Ctrl-M-3**	6.24	3.16	11.05
Ctrl-M-5	10.50	5.70	17.70
**Mean**	6.52	3.91	12.74
**SEM**	1.38	0.61	1.65
**MALT Males**			
**Malt-M-1**	5.70	4.83	12.15
**Malt-M-2**	4.61	4.04	11.05
**Malt-M-3**	5.61	4.66	12.06
**Malt-M-6**	5.67	3.50	12.12
**Mean**	5.40	4.26	11.85
**SEM**	0.31	0.35	0.31

Supplemental Table 2. Adolescent and Adult Measures of Cocaine Reinforcement under the Fixed Ratio (FR) Schedule

Monkey	Sex	Condition	Adolescent RR[1]	Adult RR[1]	Adolescent Peak Dose[2]	Adult Peak Dose[2]	Adolescent Intake[2]
**Ctrl-F-2**	Female	Control	1.19	1.80	0.01	0.003	209.00
**Ctrl-F-3**	Female	Control	1.10	0.72	0.003	0.01	75.00
**Ctrl-F-1**	Female	Control	0.52	0.34	0.03	0.01	665.00
**Malt-F-4**	Female	Maltreated	0.49	1.12	0.01	0.003	188.00
**Malt-F-2**	Female	Maltreated	1.00	0.76	0.01	0.01	254.00
**Malt-F-3**	Female	Maltreated	0.79	1.70	0.03	0.03	383.00
**Ctrl-M-3**	Male	Control	0.85	2.16	0.03	0.003	263.00
**Ctrl-M-1**	Male	Control	3.12	1.31	0.03	0.01	378.00
**Ctrl-M-2**	Male	Control	0.71	3.32	0.01	0.01	87.00
**Ctrl-M-5**	Male	Control	1.28	0.83	0.03	0.03	96.00
**Malt-M-6**	Male	Maltreated	2.26	2.74	0.01	0.03	328.00
**Malt-M-2**	Male	Maltreated	0.26	2.18	0.003	0.001	40.00
**Malt-M-1**	Male	Maltreated	0.40	1.47	0.03	0.01	415.00
**Malt-M-3**	Male	Maltreated	0.31	1.24	0.03	0.01	355.00

[1] RR: Response Rate (responses per second)
[2] Dose: mg/kg of cocaine

Supplemental Table 3. Adult Measures of Cocaine BPs under the Progressive Ratio (PR) Schedule

Monkey	Sex	Condition	Peak BP[1]	Peak BP Dose[2]
**Ctrl-F-2**	Female	Control	17.67	0.1
**Ctrl-F-3**	Female	Control	16.33	0.03
**Malt-F-4**	Female	Maltreated	16.00	0.003
**Malt-F-2**	Female	Maltreated	16.33	0.1
**Malt-F-3**	Female	Maltreated	20.00	0.03
**Ctrl-M-3**	Male	Control	16.33	0.01
**Ctrl-M-1**	Male	Control	20.00	0.03
**Cltr-M-2**	Male	Control	14.33	0.01
**Ctrl-M-5**	Male	Control	15.33	0.1
**Malt-M-6**	Male	Maltreated	15.33	0.1
**Malt-M-2**	Male	Maltreated	18.00	0.01
**Malt-M-1**	Male	Maltreated	20.00	0.1

[1] BP: peak number of injections
[2] Dose: mg/kg of cocaine

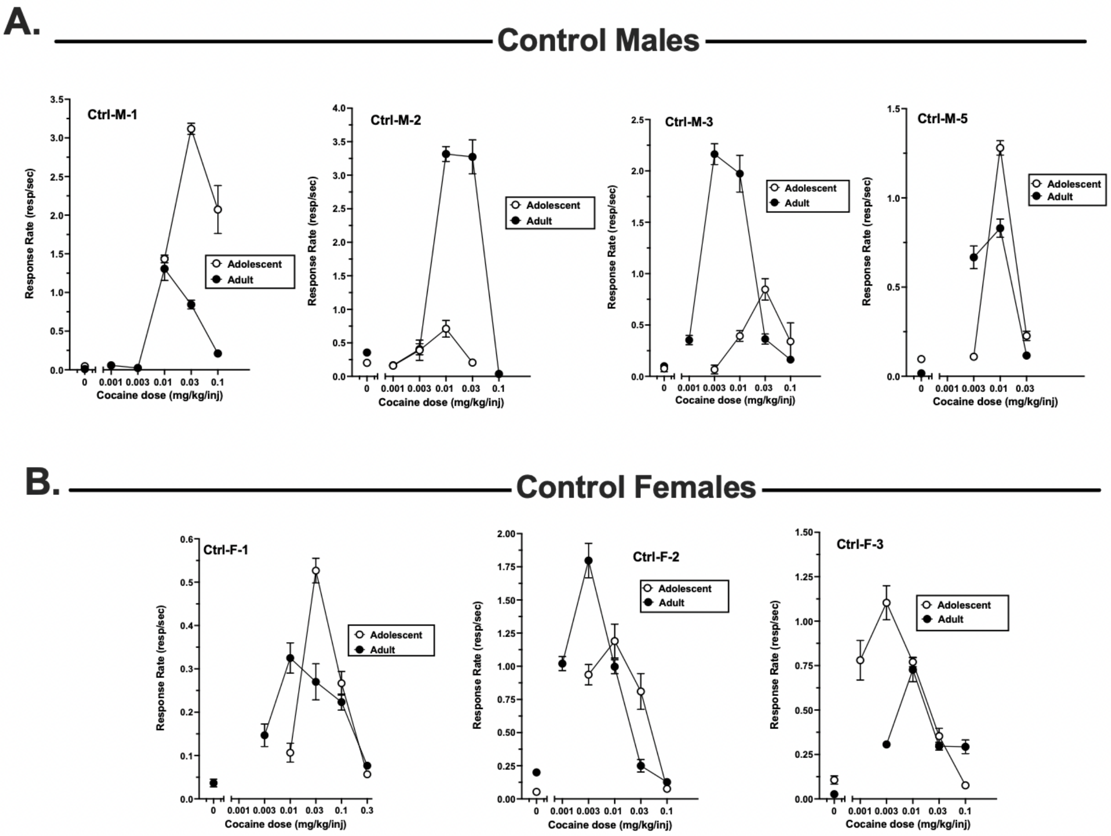
A.
Control Males
Ctrl-M-1
Ctrl-M-2
Ctrl-M-3
Ctrl-M-5
Adolescent
Adult
Response Rate (resp/sec)
Cocaine dose (mg/kg/inj)
0
0.001
0.003
0.01
0.03
0.1
0.3
B.
Control Females
Ctrl-F-1
Ctrl-F-2
Ctrl-F-3

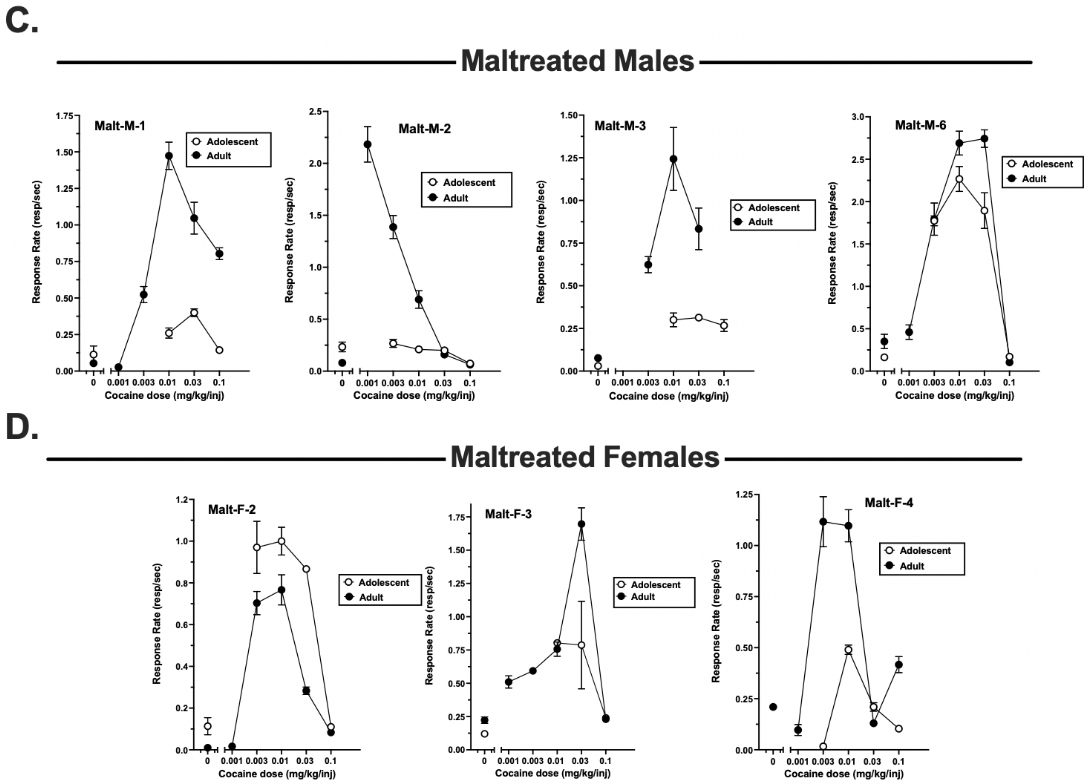

Figure S1: Rates of responding (responses per second) as a function of cocaine dose in control (panels **A** and **B**) and MALT (panels **C** and **D**) male and female rhesus monkeys. The initial cocaine dose-response curve (open circles) was previously published [1, 2]. The redetermined cocaine dose-response curves (filled circles) were generated approximately 18 months after the initial dose-response curves. Each point is the mean ± S.D. of the last 3 sessions a dose was available. N=14 (n=7 Controls, n=7 MALT)

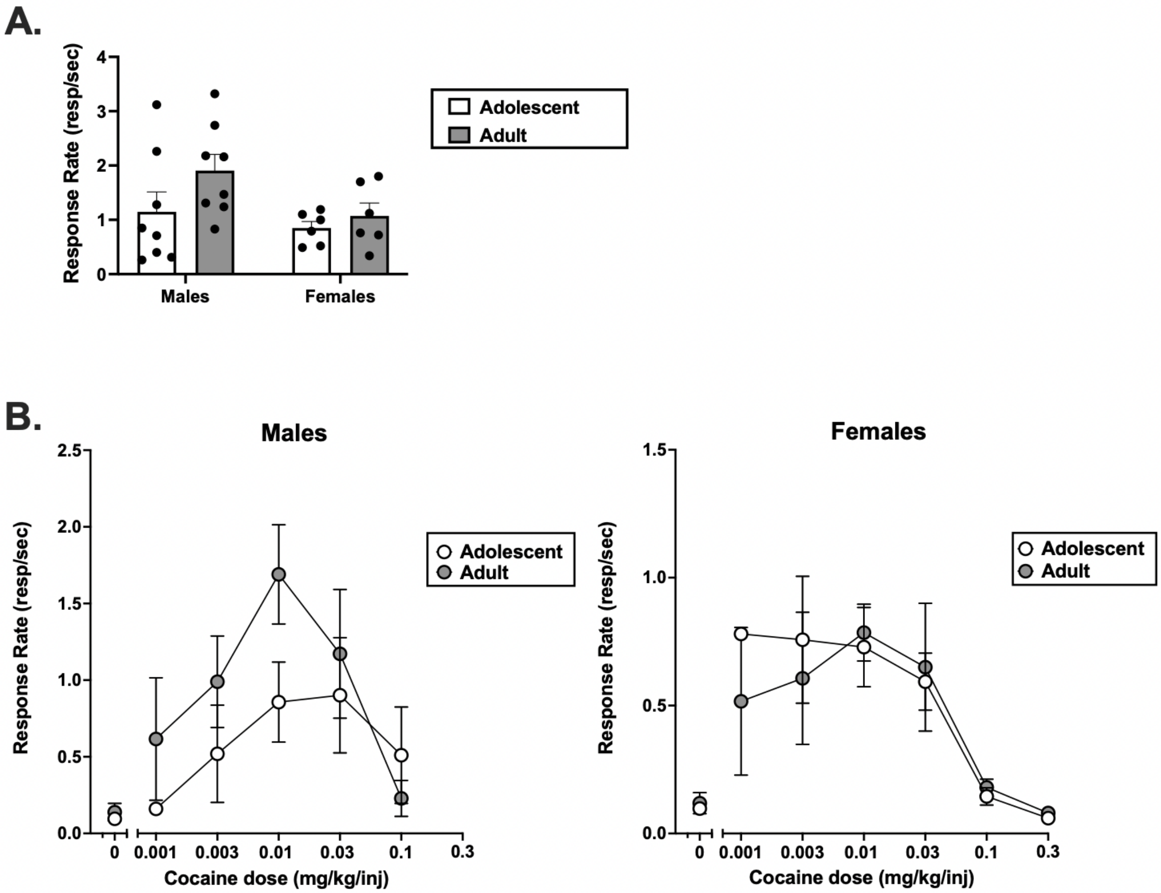

Figure S2: A: Average cocaine response rates (resp/sec), irrespective of group, at the peak of the cocaine dose-response curve in male and female monkeys during the initial determination in adolescence (white bars) and during the second determination in adulthood (black bars). **B:** Averaged dose-response curves, irrespective of group, as a function of sex (males on the left, females on the right). Data represent the mean ± the standard error of the mean. N=14 (n=8 males, n=6 females)

A.

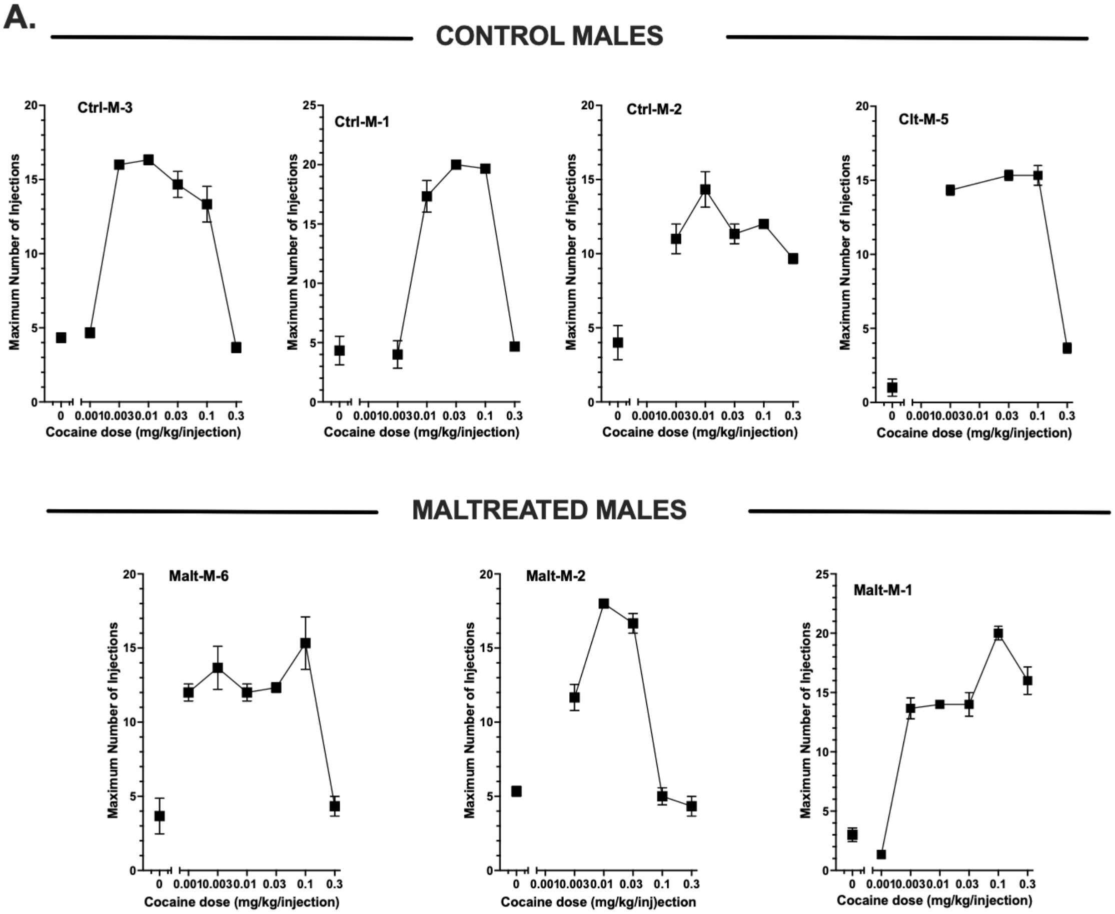
CONTROL MALES
Ctrl-M-3
Ctrl-M-1
Ctrl-M-2
Clt-M-5
MALTREATED MALES
Malt-M-6
Malt-M-2
Malt-M-1
Maximum Number of Injections
Cocaine dose (mg/kg/injection)
Cocaine dose (mg/kg/inj)ection
0
0.001
0.003
0.01
0.03
0.1
0.3

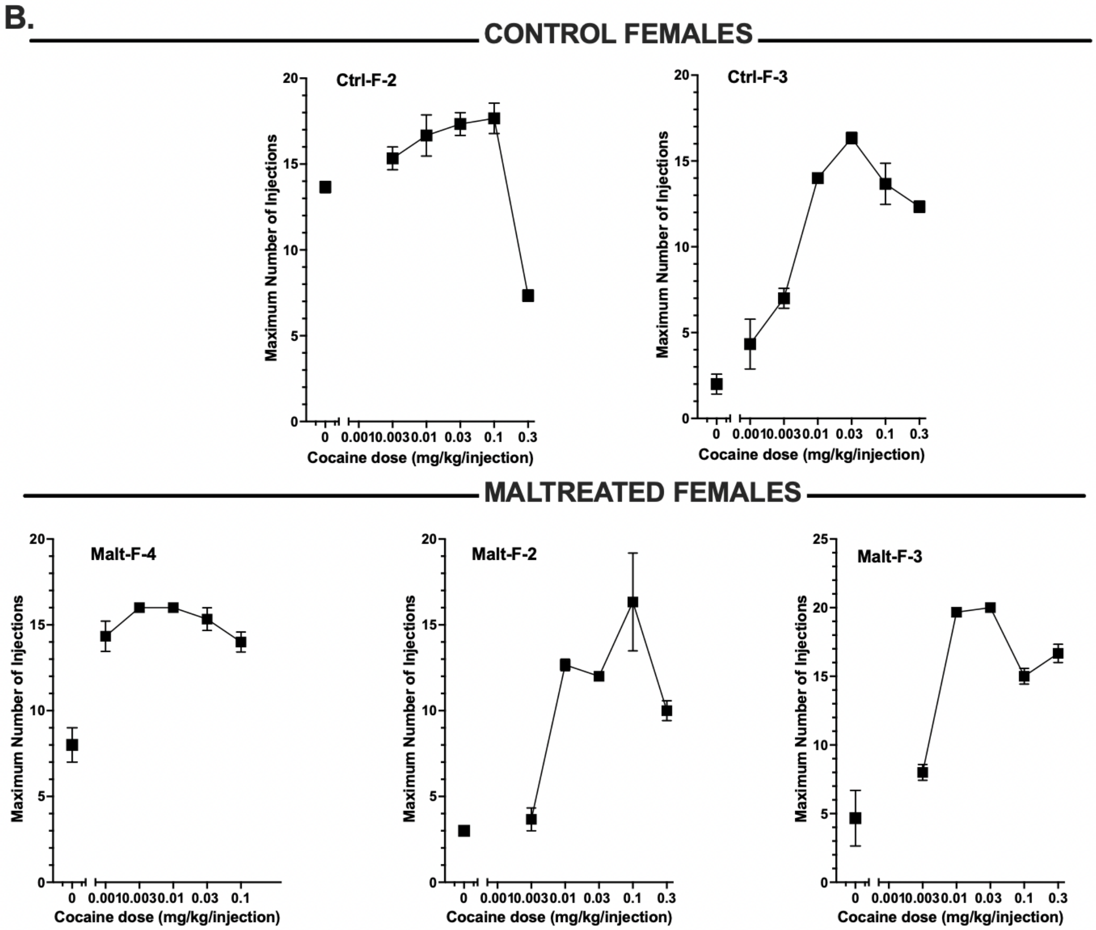

Figure S3: Maximum number of injections (break point) as a function of cocaine dose in (**A**) male and (**B**) female rhesus monkeys. Monkeys were either Controls or Maltreated as infants. Each point is the mean ± S.D. of the last 3 sessions a dose was available. N=12 (n=6 Controls, n=6 MALT)

REFERENCES

1. Wakeford, A.G.P., et al., Effects of early life stress on cocaine intake in male and female rhesus macaques. Psychopharmacology (Berl), 2020. **237**(12): p. 3583-3589.
2. Wakeford, A.G.P., et al., Effects of early life stress on cocaine self-administration in post-pubertal male and female rhesus macaques. Psychopharmacology (Berl), 2019. **236**(9): p. 2785-2796.
